# Regulation of Glucose Insulinotropic Peptide and Intestinal Glucose Transporters in the Diet-Induced Obese Mouse

**DOI:** 10.1155/2022/5636499

**Published:** 2022-02-17

**Authors:** Ryon Sun Rhodes, Satish K. Singh, Vazhaikkurichi M. Rajendran, Seth T. Walk, Steven D. Coon

**Affiliations:** ^1^Fort Peck Community College, Poplar, Montana, USA; ^2^Section of Gastroenterology, Boston University School of Medicine, USA; ^3^Section of Gastroenterology Veterans Affairs Boston Healthcare System Boston, Massachusetts, USA; ^4^Department of Biochemistry Robert C Byrd Health Sciences Center and Section of Digestive Diseases, West Virginia University School of Medicine, Morgantown, West Virginia, USA; ^5^Department of Microbiology and Cell Biology, Montana State University, Bozeman, MT, USA; ^6^Boston University Clinical and Translational Science Institute, Boston, Massachusetts, USA

## Abstract

Our recent studies have shown that glucose-dependent insulinotropic polypeptide (GIP), but not glucagon-like peptide 1 (GLP-1), augments Na-glucose transporter 1- (SGLT1-) mediated glucose absorption in mouse jejunum. Na-dependent glucose absorption sharply rose and peaked in 3 months of high-fat (i.e., obese) compared to normal (i.e., normal weight) diet fed animals. Previous studies have shown that GIP-augmented SGLT1 and PEPT1 (peptide transporter 1) are regulated by protein kinase A (PKA) signaling in mouse jejunum. Additional studies have indicated that cAMP and PI3 kinase signaling augment PEPT1 through EPAC and AKT activation pathways, respectively, through increased apical PEPT1 trafficking in intestinal epithelial cells. However, little is known about how the signaling glucose transport paradigm is altered over a long period. Early on, increased glucose absorption occurs through SGLT1, but as the obesity and diabetes progress, there is a dramatic shift towards a Na-independent mechanism. Surprisingly, at the peak of glucose absorption during the fifth month of the progression of obesity, the SGLT1 activity was severely depressed, while a Na-independent glucose absorptive process begins to appear. Since glucose transporter 2 (GLUT2) is expressed on the apical membrane of the small intestine in obese patients and animal models of obesity, it was hypothesized to be the new more efficient route. Western blot analyses and biotinylation of the apical membrane revealed that the GIP expression increases in the obese animals and its trafficking to the apical membrane increases with the GIP treatment.

## 1. Introduction

Incretins, which include glucose-dependent insulinotropic polypeptide (GIP) and glucagon-like peptide 1 (GLP-1), play important roles in the regulation of blood glucose levels by stimulating insulin release from pancreatic *β* cells during a meal [1]. Since GIP has been shown linked to nutrient absorption, it is likely that GIP may also contribute to the onset of obesity and its comorbidities such as diabetes [2–4]. As the weight of an individual increases, the increased GIP released into the intestinal tissue and vasculature resulted in stimulated nutrient absorption. Increased GIP release leads to increased insulin secretion that causes insulin insensitivity and type 2 diabetes mellitus (T2DM) ( [1, 5, 6]). GIP is also known as gastric inhibitory peptide that inhibits gastric emptying and motility. This study was performed to examine the progression of obesity and its effects on glucose absorption and investigate the paradigm shift of altered mechanism of glucose absorption at different stages during the onset of obesity.

In general, the glucose transport paradigm consists of Na-dependent glucose absorption mediated by Na-glucose cotransporter 1 (SGLT1). However, it has been shown that the glucose transporter paradigm is different in obese patients as well as obese animal models [7–11]. Although SGLT1 is expressed and shown to be functional, the Na-independent glucose transporter 2 (GLUT2) expression appears on the apical membrane of villus cells and may facilitate the increased glucose absorption in obese patients. However, the paucity of studies has not demonstrated the function of the new glucose transport paradigm sufficiently. Therefore, this study was designed to show over time the changes in the glucose absorption paradigm and how glucose absorption is altered.

Under normal conditions, carbohydrates (i.e., starch and disaccharides) are digested into monomers (e.g., glucose and fructose), which are absorbed through both Na-dependent and Na-independent pathways in the intestine [12]. Even though there are 6 SGLT isoforms, SGLT1 is the only isoform present in the intestine [13]. GLUT5, a fructose transporter, is normally coexpressed with SGLT1 on the apical membrane of villus cells of the intestinal mucosa [14]. Villus cells transport glucose and fructose monomers into villus cells, utilized by the cells to a small extent, but the bulk of these sugars are transported through the GLUT2 transporter into the portal blood. This pathway is altered in the obese individuals. As nutrients are absorbed, GIP is released to induce insulin secretion in anticipation of glucose absorption, while GIP also stimulates lipid absorption into adipocytes [1]. GIP receptors are also present in the intestinal mucosa as well as other layers of the intestine to facilitate glucose absorption [1]. GIP also feeds back to increase nutrient absorption and further increases in GIP and its effects. As a result of insulin insensitivity, GIP levels rise that increases nutrient absorption and becomes a contributing factor in the onset of obesity. The GLUT2 expression occurs on the apical membrane in obese patients as well as in obese animal models, but what triggers it and why GLUT2 is expressed is not known although most investigators speculate that GLUT2 facilitates increased glucose transport. Others in the field suggest that it may be an alternative pathway of glucose excretion in the diabetic state to reduce blood glucose levels, as metformin treatment increased GLUT2 transporters [15]. This study measured the transport of glucose through each of these two apical glucose transporters and determined their importance in the mouse model of obesity.

## 2. Methods

### 2.1. Animals

Male wild-type mice (C57/B6; 6-8 weeks old, Jackson Labs) were divided into two groups. Group 1 animals were given regular mouse chow, while group 2 animals were given a high-fat diet, which contained 47% fat by weight. Both groups were given diet and tap water *ad libitum*. Experimental protocols performed in this study were in accordance with the guidelines and regulations of both Boston University and Montana State University Institutional Animal Care and Use Committees.

### 2.2. Ussing Chamber Studies

One pair of 2 cm segments of nonstripped proximal jejunum (just distal to the ligament of Treitz) was removed from overnight fasted anesthetized mice for each experiment. The intestinal tissues were opened along the mesenteric border and mounted under voltage clamp conditions in Ussing-type chambers with 0.3 cm^2^ oblong opening (EasyMount™, Warner Instruments, New Haven, CT). Both sides of the tissue were bathed with 5 ml of Ringer's solution (in mM: 115 NaCl, 25 NaHCO_3_, 2.4 K_2_HPO_4_, 0.4 KH_2_PO_4_, 1.2 CaCl_2_, and 1.2 MgCl_2_; pH 7.4). Bathing solutions maintained at 37°C were continuously gassed with 5% CO_2_ balanced with O_2_. Following a 20 min equilibration, 10 mM O-methyl-glucose (OMG, a nonhydrolysable glucose analogue), mixed with a trace of ^3^H-3-OMG (for most experiments), was added to the mucosal bath, while 10 mM mannitol was added simultaneously to the serosal bath to maintain isoosmolarity. In other experiments, the glucose levels were determined using a glucose determination kit (Cayman Chemical). Mucosal to serosal (m-s) and serosal to mucosal (s-m) glucose fluxes, transepithelial potential difference (PD), and short circuit current (Isc; a measure of electrogenic transport process) was measured for every 15 min both in the presence and absence of incretins and inhibitors. The net glucose absorption was calculated by subtracting s-m fluxes from m-s fluxes of tissue pairs that were matched based on differences in basal conductance (G) of less than 10%. Basal conductance was calculated from the basal potential difference and short circuit current (Isc) using Ohm's law.

### 2.3. Treatments

500 *μ*M GIP (Bachem) was added to the serosal bathing solution when applicable. 10 mM phlorizen (Calbiochem) was added to the serosal bathing solution when applicable to block the action of SGLT1, respectively.

### 2.4. Western Blotting

Scraped jejunal mucosa was homogenized in RIPA buffer (Boston BioProducts, Boston, MA) containing Complete™ protease inhibitor (Roche Applied Science, Indianapolis, IN). Following a 2 hr incubation on ice, the homogenate was centrifuged at 14,000 rpm for 5 min at 4°C. 10 *μ*l of supernatant (100 *μ*g protein) collected from the homogenates was resolved on SDS-PAGE and transferred to nitrocellulose membranes (PerkinElmer, Waltham, MA). The blot was blocked two hours with 5% nonfat milk in PBST and then was incubated overnight with primary antibody (anti-GLUT2 (1 : 1000); anti-SGLT1 (1 : 1000); mouse anti-e-cadherin (1 : 2000; BD Biosciences, San Jose, CA)) at room temperature (RT) for 2 hrs. Following washes in TBST (3 × 5 min), the blot was incubated with anti-goat horseradish peroxidase-conjugated secondary antibody. Immune complexes were detected on film using enhanced chemiluminescence (SuperSignal West Pico).

### 2.5. Statistical Analyses

The values presented represent means ± SE of four tissue pairs obtained from 4 different mouse proximal jejunum. Statistical analyses were performed using unpaired or paired Student's *t*-test using GraphPad. A *p* < 0.05 is considered statistically significant.

## 3. Results

Initial time course studies on the GIP effect of glucose transport in diet-induced obese animals were performed to establish a timeline of significant changes in the routes and rates of absorption of glucose. Analysis of the data has determined changes in the normal glucose transport paradigm since new expression of different glucose transporters is involved. Short circuit current (Isc), flux measurements, and inhibition studies were made to determine the rates and possible routes of glucose absorption. Normally, glucose is absorbed by the sodium-dependent glucose transporter-1 (SGLT1), an electrogenic cotransporter of glucose and sodium through the apical membrane of intestinal villus cells. Although some glucose absorbed is phosphorylated and used by the villus cells, the bulk of glucose is then transported across the basolateral membrane into the portal blood via a facultative glucose transporter (GLUT2).

**Figure 1:**
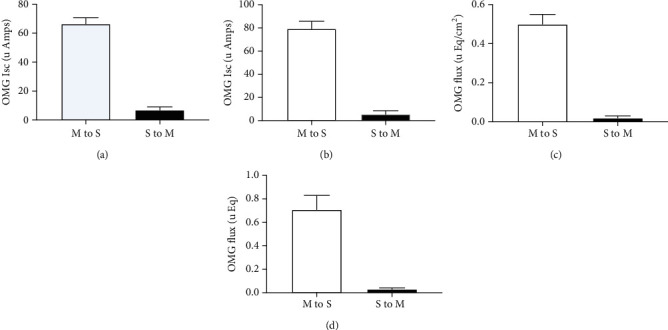
Effect of high-fat diet on SGLT1 function in mouse proximal jejunum. SGLT1 function was measured both as a function of O-methyl-glucose- (OMG-) induced Isc and as OMG fluxes in animals fed normal (a and c) or high-fat (b and d) diet. Isc was measured 45 minutes after either mucosal or serosal addition of OMG (10 mM) in normal (a) and 5 months high-fat (b) diet fed animals. To measure OMG fluxes, 10 mM OMG was added to either mucosal or serosal bath. Forty-five minutes following OMG addition, 500 *μ*l samples were taken from the opposite side to measure mucosal to serosal (m-s) and serosal to mucosal (s-m) OMG fluxes in normal (c) and 5 months high-fat (d) diet fed animals. Isoosmolality was maintained by adding 10 mM mannitol to the bath opposite to the OMG bath. Results presented represent the mean ± SE from 4 tissues obtained from 4 different mice. ^∗^*p* < 0.05—compared to basal control.

Therefore, the absorption of glucose through the SGLT1 can be measured since that generates short circuit, while sodium-independent glucose transport through the GLUTs cannot. In contrast, flux studies will measure the total amount of glucose transport through all glucose transporter proteins as well as other routes of absorption. Initially we performed experiments to determine if any significant amounts of glucose were absorbed serosal to mucosal sides of the intestine (See Figure 1). In Figures 1(a) and 1(b), the Isc is measured for both mucosal to serosal (m-s) and serosal to mucosal (s-m), and the net absorption is measured. Basal rates refer to the amount of Isc in the absence of glucose, and then, glucose was added to either the mucosal or serosal sides of the tissue to measure rates of glucose absorption in either direction in animals fed a normal and high-fat diet. This would be only the activity of SGLT1 and not GLUT2 or other transporters. The flux is measured by adding glucose to either the serosal or mucosal sides, and samples were removed from the other chamber in each case. This would be a measure of total glucose absorption including SGLT1 and GLUT2 and possibly other routes. Only in month 5, shown in Figure 1, is when the animals become obese and diabetic. In the other months, no significant amount of glucose is absorbed from the serosal side (data not shown). This data indicates that the absorption of glucose only significantly occurs from m-s and so from that point forward only those measurements were recorded.

**Figure 2:**
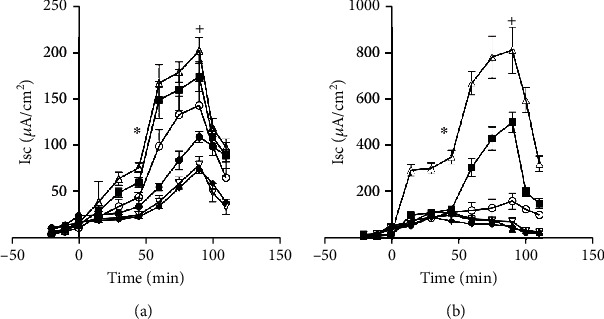
Effect of GIP on SGLT1 function in mouse proximal jejunum. SGLT1 function was measured as a function of O-methyl-glucose- (OMG-) induced Isc in animals fed a normal diet (a) and a high-fat diet (b). The 10 mM OMG-induced Isc was measured in the presence and absence of serosal GIP (0.5 *μ*M). Open circles are the initial results before animals are separated into the two diet groups (o). The first month is closed boxes (■). The second month is open triangles (*Δ*). The fifth month is closed circles (•). The seventh month is open diamonds (∇). The ninth month is closed boxes (■) Results presented represent means ± SE from 4 tissue pairs from 4 mice. ^+^*p* < 0.05—compared to normal control; ^∗^*p* < 0.05—compared to GIP control.

**Figure 3:**
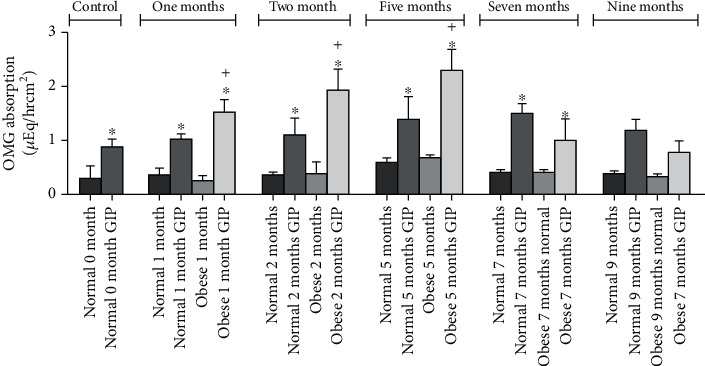
Effect of GIP on SGLT1-mediated O-methyl-glucose (OMG) absorption in normal and obese mouse proximal jejunum. Mucosal to serosal OMG absorption was measured by adding 10 mM cold OMG in mucosal bath of jejunum obtained from animals fed a normal diet (solid bars) and a high-fat diet (hatched bars). OMG absorption was also measured in the presence and absence of serosal GIP (0.5 *μ*M). Results presented represent means ± SE from 4 tissue pairs from 4 different mice. ^+^*p* < 0.05—compared to respective normal control; ^∗^*p* < 0.05—compared to respective normal control.

Jejunum isolated from normal and obese animals from various time points (after 0, 2, 5, 7, and 9 months on normal and high-fat diets) were inserted into the Ussing chambers and glucose absorption was measured in the absence and presence of GIP added to the basolateral side. Isc for each of the time points is shown in Figure 2, and the flux from the same time points is shown in Figure 3. For Figure 2, note the differences in the axis and the amount of glucose absorbed in the normal versus obese mice. In order to make the proper comparisons, the time zero experiments are presented in both figures as open circles, and these measurements were made days before any of the animals were placed on the normal or obese diets. GIP was added to the serosal side at time 45 min (^∗^), and SGLT1 activity was verified by adding a SGLT1 transport inhibitor (phlorizin) at time 90 min (+) to the mucosal side. For the normal animals, Figure 2 shows increases in the absorption via SGLT1 from the initiation of the normal diet (open circles) through the first (closed boxes) and second months (open diamonds) until the fifth month when it steeply declines (closed circles). In the obese animals, there is also a stepwise increase in absorption in the 1^st^, 2^nd^, and 3^rd^ months, but there is a substantial decrease in Isc in the 5^th^ month. Jejunum from obese animals also is more sensitive initially to GIP as compared to normal, but in later months 7^th^ and 9^th^, it appears to be totally insensitive. Not only that, in later months 7 and 9, it even appears that SGLT1 is not the route of glucose absorption because Isc is not sensitive to phlorizin.

**Figure 4:**
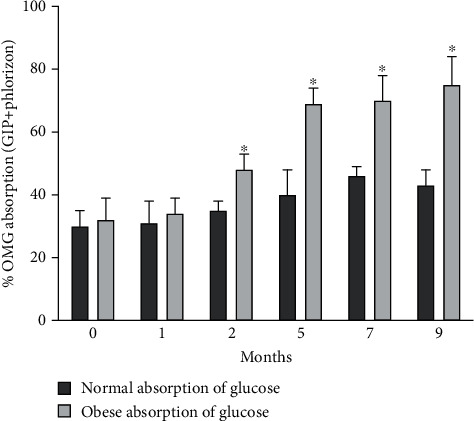
Effect of phlorizen on GIP enhanced OMG absorption in normal and obese mouse proximal jejunum. Mucosal to serosal OMG absorption was measured by adding 10 mM cold OMG in mucosal bath of jejunum obtained from animals fed a normal diet (solid bars) and a high-fat diet (hatched bars). Mucosal to serosal OMG absorption was also measured in the presence of mucosal phlorizin. Results presented represent mean ± SE from 4 tissue pairs from four different mouse. ^∗^*p* < 0.05—compared to control.

However, these results are contrary to what was observed in the flux measurements. While the Isc measurements were being made, the total flux of absorption was also measured by taking samples from the Ussing chamber baths. When glucose is added to the mucosal bath, samples are taken from the serosal bath and vice versa. These samples are taken at the 45, 90, and 110 minute time points just before the GIP and the phlorizin were added and then at the end of the experiment, respectively. Four sets of measurements are shown in Figure 2. The total amount of glucose absorbed is measured for both normal and obese tissue in the presence and absence of GIP. First, in normal mouse jejunum, although there appears to be some trend to increase slightly in the fifth month, normal absorption does not significantly change nor its sensitivity to GIP over time. However, for obese animals shown in Figure 2, flux measurements increase dramatically and significantly in the fifth month when Isc measurements have dramatically declined. This demonstrates that the rate of glucose absorption is higher but through different route(s) of absorption. What is unexpected was an overall decrease in glucose absorption in the seventh and ninth months regardless of route and transporter function. Phlorizin was used to determine the SGLT1 contribution, and the observations are shown in Figure 4. Phlorizen is an SGLT1 inhibitor that blocks the SGLT1 contribution of glucose absorption not only in Isc but also in flux measurements. In the Isc experiments shown in Figure 4, it will decrease the Isc to indicate the presence of SGLT1, and in the flux measurements, it would not only show the existence of SGLT1 function in terms of glucose absorption but also the possibility that other routes of glucose absorption exist especially in the fifth month where Isc decreased (a measure of SGLT1 function) while glucose absorption continues to increase. Since it is clear that different transporters are involved in the transport of glucose in obese animals, inhibition studies were performed. Simultaneously as the flux experiments were performed to determine the extent of SGLT1 involvement, phlorizin is added at time 90 minutes and compared to the normal control animals. As shown in Figure 4, the effectiveness of phlorizin diminishes over time starting at the 5^th^ month and continuing to the 7^th^ and 9^th^ months. This indicates that other routes of absorption become important in obesity. GLUT proteins may be expressed on the apical membranes of jejunum isolated from obese animals to facilitate glucose.

**Figure 5:**
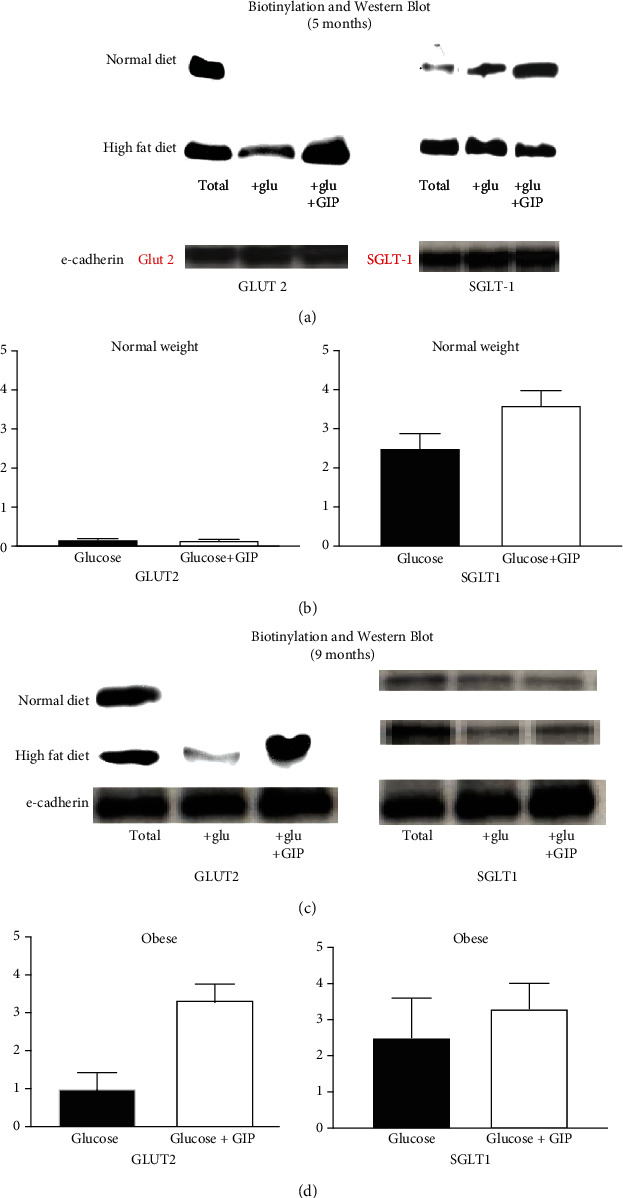
SGLT1 and GLUT2-specific protein expression in animals fed a normal and high-fat diet after 5 months (a and b) and 9 months (c and d). (a and c) Biotinylation and western blots of GLUT2 (a) and SGLT1 (b) proteins are shown. Total lanes are total cell homogenates (no biotinylation) that includes both the apical, basolateral, and internal membranes of villus cells and serves as a control. Jejunum from animals at five months of a normal and high-fat diet are used. Each glucose (+glu) and glucose and GIP (+glu+GIP) blots represent cell homogenates prepared from biotinylations from jejunal mucosa along with a total homogenate each from a single animal. Since actin is not present in the membranes, e-cadherin loading controls are used instead. A representative blot is shown from 4 different animals. (b and d) Densitometry of 4 representative blots is shown. Results presented represent means ± SE from four mouse jejunums. ^∗^*p* < 0.05—compared to control. ^+^*p* < 0.05—compared to control.

In order to better determine a possible mechanism for the increased glucose absorption, biotinylation and subsequent western blotting experiments were performed. These experiments were performed at 5 months (Figures 5(a) and 5(b)) and at 9 months (Figures 5(c) and 5(d)). In Figure 5, panel (a) is a typical blot for each month, while in panel (b), the densitometry of 4 typical blots is shown. Biotinylation will isolate only those proteins on the apical membrane. At 5 months, there is a switch in the glucose absorption paradigm, and whether that changes as the glucose absorption changes in the seventh and ninth months is shown in the biotinylation/western blots performed with animals from normal and obese animals. Since GLUT2 proteins are expressed on the basolateral membrane, biotinylation is necessary before western blotting can be performed to show if any expression occurs on the brush border. Additional controls were used to ensure the purity of the samples by doing western blots on PEPT1 (present only on the apical membrane, data not shown) and NaKATPase (present only on the basolateral membrane but absent on the apical membrane). The presence of the NaKATPase will indicate that there is basolateral membrane contamination (data not shown) as well as using e-cadherin as a loading control since actin is not present in brush border membrane.

Total proteins shown are from total homogenates and not from any biotinylations. This shows that in both normal and high-fat diets, SGLT1 and GLUT2 are present somewhere in cellular membranes. For SGLT1 protein, in normal weight animals, GIP increases apical expression of SGLT1, but for obese jejunum, such an increase by GIP does not occur in either month five or month nine (Figure 5). SGLT1 protein expression and is response to GIP appears to decrease during obesity at nine months. It is no longer statistically significant.

For GLUT2 proteins however, GLUT2 is not present in the apical membranes isolated from normal jejunum, but in obese animals, GLUT2 is not only present, but also its expression on the apical membrane increases with GIP treatment. This suggests significant shifts in the glucose transport paradigm that alters the absorption of glucose and the sensitivity of these transporters to incretins such as GIP. What is unexpected is that there is little change in the responses and expression of any of the glucose transporters in month nine as shown in Figures 5(c) and 5(d). The amount of glucose transport declines significantly, yet the expression and sensitivity to GIP remain the same. Although Ussing chamber Isc studies did not indicate a reversal of glucose transport through SGLT1, it is possible that a reversal or reduction of glucose transport through GLUT2 may occur as a result of diabetes and high blood glucose levels. However, the mechanism of such a change is not evident using our experimental design.

## 4. Discussion

This study shows for the first time how obesity influences the normal paradigm of glucose absorption. SGLT1 is expressed on the apical membrane of villus cells in the intestinal mucosa of both normal and obese animals but transports less of the glucose load and becomes less responsive to GIP as the animals become more obese. GLUT2, only present on the basolateral membrane of villus cells in normal villus cells of mouse intestine, is expressed on both the basolateral and the apical membrane as the animals become obese. GLUT2 functions to initially increase glucose absorption but has a declining function as the animals become morbidly obese and diabetic.

The overall role of GIP in the absorption of nutrients has just recently been investigated. GIP has long been known to be secreted when nutrients are absorbed in the intestine [1], and just recently, GIP was demonstrated to increase the absorption of nutrients such as glucose [16] and peptides [17] in the normal intestine. This would suggest that GIP works in a cyclic manner to increase nutrient absorption and its own secretion to eventually regulate insulin secretion and ultimately control blood glucose levels. This perpetuating cycle may also explain how the overabsorption of nutrients might increase GIP to such levels that it would overstimulate insulin secretion that would potentially cause insensitivity and diabetes.

In our study, disparity between the results of the Isc measurements and the overall flux of glucose absorption is quite significant. The role of overall glucose absorption (flux) in the normal mouse remains relatively constant throughout its lifetime, and its response to GIP seems to be stable as well through the first nine months on the normal diet although there may be some trend towards an increase around the fifth month. This may perhaps in response to increasing growth and maturation into adulthood. Nevertheless, no significant changes occur to the overall absorption of glucose in the mouse jejunum. In contrast, for obese animals, the glucose absorption overall does not significantly change but the sensitivity dramatically increases. The flux data demonstrates that the absorption of glucose changes little from normal to obese, but the reason for increase in glucose is solely due to the effect of GIP on glucose absorption. What is unusual is the fact that this response decreases after the fifth month and in fact decreases eventually below the normal controls.

In normal animals, the glucose transport paradigm is solely dependent on SGLT1. In our study, SGLT1 has been demonstrated to be the major glucose transporter in normal jejunum and appears to be the only electrogenic glucose transporter expressed on the apical membrane of intestinal villus cells since phlorizin blocks the Isc. As the animals get older, the amount of glucose absorbed increases initially but eventually decreases steadily, yet the response is still sensitive to GIP. Why the overall flux of glucose absorption does not decrease as well and is relatively unchanged seems to indicate that other routes of glucose absorption may be involved in normal jejunum even though that seems unlikely. There may be other GLUT proteins present even in normal, or perhaps some small amounts of glucose can be transported through GLUT5 that is normally on the normal apical membrane. Biotinylation and western blotting show that GLUT2 is not expressed in the apical membrane of normal villus cells, so if there is an alternate route, it still needs to be determined. Nevertheless, decreases in the GIP sensitivity do occur in both the Isc measurements and the total flux of glucose absorption.

However, in obese animals, the functional role of SGLT1 differs significantly. SGLT1 activity also initially rises and then declines over the following months just as in normal animals but at a much greater extent. In addition, by month five, its function nearly disappears contrary to what is observed in the normal animal and becomes now insensitive to GIP. Since glucose transport through SGLT1 appears to decrease, this demonstrates that a new major mechanism must account for absorption of glucose other than SGLT1. It has been recently shown that GLUT2 is also expressed eventually on the apical membrane of villus cells to presumably facilitate increased absorption of glucose but that has not been proven conclusively ( [18, 19]). In this study, we demonstrated that there is a dramatic increase in glucose absorption and then an eventual decrease. The increase early on is due to increased SGLT1 activity that later is replaced by GLUT2 as the increases in glucose absorption rise. However eventually with both SGLT1 and GLUT2 present, glucose absorption decreases. This was an unexpected result since it was anticipated that with decreased glucose absorption, decreases in apical GLUT2 expression would probably occur, yet expression levels of GLUT2 and SGLT1 remained unchanged. This may suggest another route of glucose absorption that decreases as obesity and diabetes progress.

Since the GLUT2 expression is altered in villus cells (9; 19), biotinylation and western blotting targeted this protein as a possible additional route of glucose absorption in villus cells. However, it may not be the only one. Other GLUT proteins or other transporters may also be involved and should not be discounted. Figure 3 shows that SGLT1 activity declines in the fifth month and beyond, but further studies utilizing inhibitors to the GLUTs will be necessary to further delineate the glucose routes of absorption.

GIP effects on SGLT1 and GLUT2 are quite profound by an increase in the apical expression of these transporters more likely by an increase in trafficking of these proteins from intracellular stores. Because the response is less than an hour, trafficking of the SGLT1 and GLUT2 transporters is the most likely mechanism. In normal jejunum where only SGLT1 is present, it is trafficked to the membrane upon treatment with GIP (at five months or nine months). However, in the obese animals, SGLT1 is unaffected by GIP but GLUT2 is trafficked to the apical membrane instead. What is unexpected is that even in the ninth month, the expression of GLUT2 and its GIP response does not appear to change despite decreases in both the Isc and the flux of glucose. Recent studies have shown that during the diabetic state, less glucose is absorbed and perhaps even excreted as a response to increasing blood glucose levels. However, serosal to mucosal transport of glucose in our study did not rise to significant levels to support such a conclusion (data not shown). Therefore, it would suggest that GIP as well as glucose insensitivity becomes an issue in the diabetic state.

## Data Availability

All original data can be found with the corresponding author.
